# MANF: A New Player in the Control of Energy Homeostasis, and Beyond

**DOI:** 10.3389/fphys.2018.01725

**Published:** 2018-11-29

**Authors:** Su Yang, Shihua Li, Xiao-Jiang Li

**Affiliations:** ^1^Department of Human Genetics, Emory University School of Medicine, Atlanta, GA, United States; ^2^GHM Institute of CNS Regeneration, Jinan University Guangzhou, China

**Keywords:** MANF, energy homeostasis, neurotrophic factors, mouse models, neurodegeneration

## Abstract

All human behaviors, including the control of energy homeostasis, are ultimately mediated by neuronal activities in the brain. Neurotrophic factors represent a protein family that plays important roles in regulating neuronal development, function, and survival. It has been well established that canonical neurotrophic factors, such as brain-derived neurotrophic factor (BDNF) and ciliary neurotrophic factor (CNTF), play important roles in the central regulation of energy homeostasis. Recently, a class of non-canonical neurotrophic factors, represented by mesencephalic astrocyte-derived neurotrophic factor (MANF), has been discovered. MANF is structurally and functionally distinct from those canonical neurotrophic factors, hence raising the issue of MANF being non-canonical. Nonetheless, emerging evidence suggests that MANF is critically involved in many neuronal activities. Here, we review our current understanding about the functions of MANF in the brain, with a primary focus on the control of energy homeostasis.

## Main

Obesity is a global pandemic affecting both children and adults. According to Institute for Health Metrics and Evaluation database, 2.1 billion people, or 29% of the world’s population, were either overweight or obese in 2013. Obesity is a risk factor for cardiovascular diseases, stroke, type 2 diabetes, high blood pressure and certain types of cancer (Poirier et al., 2006), which imposes profound economic and health care burdens on the individual and society. Genetic factors are estimated to account for 67% of variance in body weight and human adiposity (Maes et al., 1997). More than 150 genetic loci have been associated with the development of obesity, yet these loci only explain 2% of overall obesity cases (Drong et al., 2012), highlighting an urgent need to expand our knowledge of the molecular mechanisms leading to obesity.

Obesity arises due to unbalanced energy intake and expenditure, both of which are critically controlled by a complex system that is comprised of different organs including the liver, pancreas, muscle, adipose tissues, gastrointestinal tract and brain. Within the system, the brain serves as a pivotal hub for information integration and processing, as different signals generated by the peripheral tissues, including leptin produced in the adipose tissues (Zhang et al., 1994), insulin produced by the pancreas (Woods et al., 1979; Baskin et al., 1999), ghrelin produced by the stomach (Nakazato et al., 2001), glucagon-like peptide 1 (GLP-1) and peptide YY 3-36 (PYY_3-36_) produced by the intestine (Turton et al., 1996; Batterham et al., 2002). These peripheral signals converge into specific brain regions such as the hypothalamus and brainstem (Schwartz et al., 2000; Wynne et al., 2005; Morton et al., 2006; Schwartz, 2010), which function to change neuronal activities in these regions to regulate energy intake and expenditure.

Neuronal activities are not merely controlled by secreted factors from peripheral tissues. Neurotrophic factors, which are secreted proteins synthesized locally in the brain, signal through their respective transmembrane receptors and act upon various neuronal populations. Neurotrophic factors are functionally versatile, which facilitate neuronal growth, differentiation, survival, synaptic formation and plasticity during development, as well as in the mature brain (McAllister et al., 1999; Huang and Reichardt, 2001; Vicario-Abejon et al., 2002). To date, two types of neurotrophic factors, including brain-derived neurotrophic factor (BDNF) and ciliary neurotrophic factor (CNTF), have been linked to the central control of energy homeostasis, as mutations in the genes encoding BDNF and its receptor tropomyosin receptor kinase B (TrkB) are found in patients with severe obesity (Yeo et al., 2004; Gray et al., 2006; Han et al., 2008), and administration of CNTF protein leads to body weight loss in both human and mice (Miller et al., 1996; Gloaguen et al., 1997). Considering their important and diverse roles in the brain, it is likely that additional neurotrophic factors are involved in the regulation of energy homeostasis, so that this process can be manipulated in a highly precise and regulated manner.

Indeed, emerging studies suggest that mesencephalic astrocyte-derived neurotrophic factor (MANF), a recently identified neurotrophic factor, could also serve as a regulator of food intake and body weight. Given that both the protein structure and functional mechanisms of MANF are different from most other neurotrophic factors, whether MANF is truly a neurotrophic factor remains a matter of debate. In this review, we refer MANF as a non-canonical neurotrophic factor. We begin with an overview of MANF as a neuroprotective molecule; we then present the recent evidence supporting the role of MANF in mediating energy homeostasis and compare MANF with BDNF and CNTF, the two neurotrophic factors that have been extensively studied; we also discuss the latest research about the endogenous functions of MANF in the brain, and argue that besides promoting neuronal survival, MANF could possess additional roles in mediating neuronal development and activities.

## Manf: a New Addition to the Neurotrophic Factor Family

Mesencephalic astrocyte-derived neurotrophic factor was initially discovered in the conditioned medium from ventral mesencephalic cell line 1 (VMCL1), in 2003 (Petrova et al., 2003). Being considered as a non-canonical neurotrophic factor, MANF exhibits several major distinctions comparing to other previously known neurotrophic factors: (1) most canonical neurotrophic factors evolved early in vertebrate history, whereas MANF represents a much more ancient protein species, as MANF homologs are found in invertebrates, such as fruit fly *Drosophila melanogaster* (Palgi et al., 2009), nematode *Caenorhabditis elegans* (Bai et al., 2018) and sponge *Suberites domuncula* (Sereno et al., 2017); (2) MANF does not share any protein sequence homology with canonical neurotrophic factors. In fact, the N-terminus of MANF is homologous to saposin-like proteins (Parkash et al., 2009), and the C-terminus resembles SAF-A/B, Acinus and PIAS (SAP) proteins (Hellman et al., 2011), suggesting two distinct functions (Lindahl et al., 2017); (3) like canonical neurotrophic factors, MANF is able to work extracellularly to regulate cellular signaling cascades (Yang S. et al., 2014; Zhang et al., 2017a,b; Tseng et al., 2018), potentially through an unidentified transmembrane receptor. But more intriguingly, MANF is also localized intracellularly in the endoplasmic reticulum (ER), and functions as an ER stress response protein (Mizobuchi et al., 2007; Apostolou et al., 2008). In fact, through interacting with glucose-regulated protein 78 (GRP78), an ER chaperone, most of intracellular MANF is retained in the lumen of ER (Oh-Hashi et al., 2012). Upon ER stress, the interaction is attenuated, and MANF is released to the extracellular space (Glembotski et al., 2012). The dual functional locations make MANF a unique target for research, which may shed light on novel therapeutic strategies that are previously inaccessible to canonical neurotrophic factors.

Early functional studies about MANF are focused on its role in Parkinson’s disease (PD), as genetic knockdown or knockout of *manf* in zebrafish or fruit fly lead to defective development of the dopamine system (Palgi et al., 2009; Chen et al., 2012), and administration of purified MANF protein or viral vectors expressing MANF has beneficial effects in both cellular and animal models of PD (Petrova et al., 2003; Zhou et al., 2006; Voutilainen et al., 2009; Hao et al., 2017). Endogenous MANF protein is abundantly expressed in the rodent brain, as well as non-neuronal tissues, such as liver, salivary gland and testis (Lindholm et al., 2008). Emerging evidence indicates that MANF is protective in a broad range of disease conditions, including cerebral ischemia (Airavaara et al., 2009; Yu et al., 2010; Matlik et al., 2018), myocardial infarction (Glembotski et al., 2012), Spinocerebellar ataxia type 17 (Yang S. et al., 2014; Guo et al., 2018), and retinal degeneration (Neves et al., 2016; Lu et al., 2018). Although the exact mechanism remains elusive, both extracellular and intracellular MANF forms are believed to contribute to the protective effects. Several lines of evidence support the extracellular functions of MANF: in cultured cells, addition of recombinant MANF protein into the culture medium is able to activate pro-survival signaling pathways, including PKC, AKT/GSK3β, AMPK/mTOR and STAT3 pathways (Yang S. et al., 2014; Zhang et al., 2017a,b; Tseng et al., 2018); in fruit fly and mouse, administration of recombinant MANF protein ameliorated retinal degeneration caused by various damaging stimuli, and the protective capacity could be derived from immune modulation (induction of alternative activation of microglia) (Neves et al., 2016); in rodent models of PD and stroke, administration of recombinant MANF protein reduced neuronal death (Airavaara et al., 2009; Voutilainen et al., 2009; Yang W. et al., 2014). A major obstacle to advance our knowledge about extracellular MANF is that the identity of its plasma membrane receptor remains unknown, although different mechanisms have been proposed to account for the cellular uptake of MANF (Henderson et al., 2013; Bai et al., 2018). There are also several studies supporting the intracellular functions of MANF: MANF is enriched in the ER and is induced by unfolded protein response (UPR) (Mizobuchi et al., 2007; Apostolou et al., 2008); upon UPR and ER stress, MANF could be either secreted to the extracellular space (Oh-Hashi et al., 2012; Hartley et al., 2013), or enter the nucleus to suppress the transcriptional activities of NF-κB pathway (Chen et al., 2015).

## Manf as a Regulator of Energy Homeostasis

Energy homeostasis is achieved by balanced energy intake and expenditure. As the first neurotrophic factor known to be involved in the control of energy homeostasis, BDNF is believed to play a crucial role in both processes. Two-day food restriction was able to selectively reduce *Bdnf* mRNA level in the ventromedial nucleus of hypothalamus (VMH) in mice (Xu et al., 2003), an area known to regulate energy intake. Intracerebroventricular infusion of recombinant human BDNF decreased food intake in rats (Lapchak and Hefti, 1992), whereas genetic deletion of *Bdnf* gene in the VMH or the paraventricular nucleus of hypothalamus (PVH), another brain area related to energy intake, led to hyperphagia and obesity in mice (Unger et al., 2007; An et al., 2015). Nonetheless, it remains to be determined where the BDNF expressing neurons project to, and how BDNF functions to suppress food intake. On the other hand, injection of BDNF protein into either the VMH or PVH increased energy expenditure in rats, via escalating heat production and resting metabolic rate (Wang et al., 2007, 2010). These results are further corroborated by the finding that deletion of *Bdnf* gene in the PVH reduced energy expenditure in mice, via decreasing locomotor activity and thermogenesis (An et al., 2015). Again, the neural circuits that mediate the effect of BDNF on energy expenditure remain to be elucidated, although it has been found that the BDNF expressing neurons in the medial and posterior PVH could project to the spinal cord and promote adaptive thermogenesis through polysynaptic connections to brown adipose tissues (An et al., 2015). Taken together, BDNF functions to counteract obesity through reducing energy intake and enhancing energy expenditure.

Ciliary neurotrophic factor belongs to the interleukin 6 (IL-6) cytokine family (Heinrich et al., 2003). Originally discovered as a pro-survival factor for chick ciliary ganglion neurons (Adler et al., 1979), CNTF mRNA and protein are not only expressed in neuronal tissues, but also widely distributed in non-neuronal tissues, including heart, lung, liver, kidney and testis (Ohta et al., 1995, 1996). Similar to MANF, CNTF is localized intracellularly, and is secreted to the extracellular space during cell injury (Lin et al., 1989; Stockli et al., 1989; Rudge et al., 1992). CNTF is essential for the survival of motor neurons (Masu et al., 1993), hence has been tested as a therapy for amyotrophic lateral sclerosis (ALS). However, quite unexpectedly, CNTF treatment in ALS patients led to marked body weight loss (Miller et al., 1996). This phenomenon has been reproduced in mice, as systemic administration of CNTF reduced hyperphagia and obesity in mice deficient with functional leptin (ob/ob mice) or leptin receptor (db/db mouse) (Gloaguen et al., 1997). The mechanism underlying the effect of CNTF is believed to be partially from the leptin signaling, as both leptin and CNTF activate the Janus tyrosine kinase–signal transducers and activators of transcription (JAK–STAT) signaling (Vaisse et al., 1996; Peterson et al., 2000), especially the STAT3 transcription factor, which is a key molecule involved in energy balance (Bates et al., 2003). Nonetheless, null mutations of *Cntf* were not associated with either hyperphagia or obesity in mice or human (Takahashi et al., 1994; DeChiara et al., 1995), indicating that the endogenous function of CNTF might not be related to energy homeostasis.

**FIGURE 1:**
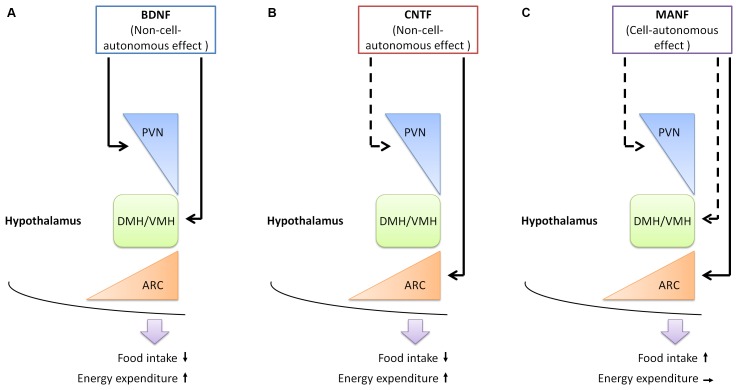
The functions of neurotrophic factors in the hypothalamus. **(A)** BDNF expressing neurons are found in the PVN, DMH, and VMH of the hypothalamus. BDNF functions in these regions to reduce food intake and increase energy expenditure, in a non-cell-autonomous manner. **(B)** The receptor for CNTF is found in the PVN and ARC of the hypothalamus. Administration of CNTF alters neuronal activities in the ARC, but not in the PVN. The outcome of CNTF administration is reducing food intake and increasing energy expenditure. **(C)** MANF is expressed in the PVN, DMH, VMH, and ARC of the hypothalamus. Increasing MANF expression in the hypothalamus, or specifically in the ARC leads to increased food intake, in a cell-autonomous manner, as administration of recombinant MANF protein does not change food intake. The function of MANF in other regions of the hypothalamus remains to be established. Arrows with solid lines, experimental evidence is currently available; arrows with dotted lines, experimental evidence is currently lacking. PVN, paraventricular hypothalamus; DMH, dorsomedial hypothalamus; VMH, ventromedial hypothalamus; ARC, arcuate nucleus.

Compared with the neurotrophic factors described above, evidence supporting the involvement of MANF in energy homeostasis is only beginning to emerge (Figure 1 and Table 1). MANF is abundantly expressed in the brain (Lindholm et al., 2008). In rats, the expression of MANF is particularly high during early development (between postnatal day 3 and 5) in the brain, but gradually declines as the brain matures. Nonetheless, high expression of MANF sustains into adulthood in selected brain regions, including hypothalamus, the brain region that crucially regulates energy homeostasis (Wang et al., 2014). Interestingly, an exome sequencing study identified *MANF* as a potential causative gene for a 22-year-old woman patient with type 2 diabetes mellitus, hypothyroidism, primary hypogonadism, short stature, mild intellectual disability, obesity, deafness, high myopia, microcephaly and partial alopecia (Yavarna et al., 2015). More clinical data are needed to firmly establish the link between MANF and obesity development in human.

**Table 1:** Comparison of BDNF, CNTF, and MANF in energy homeostasis.

Name	Protein structure	Expression pattern	Overall function in energy homeostasis	Important studies/findings
BDNF	N-terminal signal peptide; followed by a common, basic consensus sequence of the furin type; C-terminal conserved sequence of nerve growth factor (NGF) domain.	High expression in the brain, low expression in non-neuronal tissues.	Reduce food intake, increase energy expenditure.	BDNF is enriched in the ventromedial hypothalamus (VMH); brain administration of BDNF reduces food intake (Xu et al., 2003). Deletion of BDNF in the VMH and dorsomedial hypothalamus (DMH) leads to hyperphagia (Unger et al., 2007).
				Deletion of BDNF in the paraventricular hypothalamus (PVH) leads to hyperphagia and reduced energy expenditure (An et al., 2015).
				
CNTF	Lacks a signal peptide; resembles alpha-helical cytokine family which is characterized by a bundle of four anti-parallel helices.	High expression in the brain, heart, lung, liver, kidney and testis.	Reduce food intake, increase energy expenditure.	Brain administration of CNTF reduces food intake and adiposity (Gloaguen et al., 1997). Germline deletion of CNTF does not lead to hyperphagia or obesity (Masu et al., 1993).
				
MANF	N-terminal signal peptide; followed by a domain homologous to saposin-like proteins (SAPLIPs); C-terminus homologous to SAF-A/B, Acinus and PIAS (SAP) protein superfamily.	High expression in the brain, liver, salivary gland and testis.	Increase food intake, no effect on energy expenditure.	MANF is enriched in the ARC, VMH and PVH; deletion of MANF in the hypothalamus leads to reduced food intake; overexpression of MANF in the hypothalamus leads to hyperphagia (Yang S. et al., 2014).


The most direct support for the role of MANF in controlling energy homeostasis comes from a recent study on mice (Yang et al., 2017): MANF was found to be enriched in different nuclei in mouse hypothalamus, including the arcuate nucleus (ARC), VMH and PVH; two-day food restriction significantly increased MANF expression in the hypothalamus; increasing or decreasing MANF expression selectively in the hypothalamus led to hyperphagia or hypophagia, respectively. MANF appeared to specifically regulate energy intake, while leaving energy expenditure unaffected. In terms of mechanistic studies, MANF was shown to function in the ER to mediate insulin response, a signaling pathway known to regulate energy homeostasis in the brain (Vogt and Bruning, 2013), via influencing the activity of a kinase named PIP4k2b. Taken together, these results demonstrate that the activity of MANF leads to enhanced energy intake and functions opposite to BDNF and CNTF. Nonetheless, there remain several open questions that are critical for elucidating the exact role of MANF in the hypothalamus: what is the specific function of MANF in each hypothalamic region or neuronal type; are there any other intracellular or extracellular mechanisms that account for the hyperphagic effect of MANF? It has been well established that obesity could cause ER stress (Ozcan et al., 2004), and hypothalamic ER stress leads to leptin/insulin resistance and hyperphagia (Zhang et al., 2008; Ozcan et al., 2009). Given that MANF protects against ER stress, it appears counterintuitive that increased MANF expression in the hypothalamus is associated with hyperphagia. Nonetheless, in most cases, the up-regulation of MANF during ER stress was caused by acute treatment, such as ER stress inducing chemicals, or ischemic and epileptic insults (Apostolou et al., 2008; Lindholm et al., 2008). It remains unknown whether MANF is up-regulated under chronic ER stress conditions, such as during the development of obesity.

It is worth noting that MANF seems to be closely linked to the insulin signaling, as germline knockout of *Manf* in mice led to a progressive reduction of pancreatic β cells, the type of cell responsible for the synthesis and storage of insulin (Lindahl et al., 2014). On the other hand, addition of recombinant MANF protein was able to protect human pancreatic β cells against stress-induced cell death (Hakonen et al., 2018). Homozygous *Manf* knockout mice showed retarded body growth, which is in agreement with the obesity phenotype found in a transgenic mouse model overexpressing MANF (Yang et al., 2017). Intriguingly, brain specific knockout of *Manf* during early development, which is mediated by Cre recombinase driven by *Nestin* promoter, did not result in any obvious phenotypes (Lindahl et al., 2014). It is possible that certain compensatory mechanisms exist during development. Indeed, a similar phenomenon was found in BDNF studies: deletion of *Bdnf* in the adult PVH caused much more robust hyperphagia and obesity than deletion of Bdnf in the PVH during embryonic development using Cre recombinase driven by *Sim1* promoter (Balthasar et al., 2005; An et al., 2015).

## Conclusion and Future Perspectives

As discussed above, researchers have begun to reveal the potential roles of MANF in mediating energy homeostasis. The functional mechanisms of MANF remain largely unclear. Especially, the dual modes of action both inside and outside of the cell add an additional layer of complexity, which at the same time, offer an exciting research opportunity. It is highly desirable to identify the plasma membrane receptor for MANF, so that we could get a clear picture about the signaling pathways controlled by MANF. It is equally important to clarify the intracellular molecular mechanisms of MANF, not only in the ER, but potentially in other cellular organelles or structures. In addition, identification of the neuronal types and the related neural circuits that MANF acts upon would provide much needed information not only to expand our understanding of the central control of energy homeostasis, but also to design therapeutic approaches to combat the development of obesity. MANF is widely distributed throughout the brain (Lindholm et al., 2008; Yang et al., 2017), and it affords protection to neurons in different brain regions, such as the cortex, cerebellum and substantia nigra (Voutilainen et al., 2009; Yang S. et al., 2014; Tseng et al., 2018). Notably, the expression of MANF appears to be higher in the developing brain (Wang et al., 2014). These results suggest that MANF could possess important cell type-dependent functions in brain regions other than the hypothalamus, especially during early development. Indeed, a recent study indicates that MANF could promote neurite extension and neuronal migration in the developing cortex (Tseng et al., 2017). Continual efforts are needed for us to build a comprehensive image about the neurobiology of MANF in the brain.

## Author Contributions

SY wrote the manuscript. SY, SL, and X-JL discussed and revised the manuscript. All authors read and approved the final version of the manuscript.

## Conflict of Interest Statement

The authors declare that the research was conducted in the absence of any commercial or financial relationships that could be construed as a potential conflict of interest.
